# Molecular Detection and Characterization of Tick-Borne Pathogens in *Ixodes ricinus* Ticks Collected from Humans

**DOI:** 10.3390/pathogens14060528

**Published:** 2025-05-25

**Authors:** Marina Žekić, Tjaša Cerar Kišek, Vesna Cvitković-Špik, Eva Ružić-Sabljić, Vladimir Gajdov, Aleksandar Potkonjak, Aleksandar Jurišić, Sara Savić

**Affiliations:** 1 Scientific Veterinary Institute “Novi Sad”, 21113 Novi Sad, Serbia; vladmir.g@niv.ns.ac.rs (V.G.); sara@niv.ns.ac.rs (S.S.); 2 National Laboratory for Health, Environment and Food, Department for Public Health Microbiology, SI-1000 Ljubljana, Slovenia; tjasa.cerar.kisek@nlzoh.si; 3 Laboratory for Diagnostics of Borreliosis and Lepotospirosis, Institute of Microbiology and Immunology, Faculty of Medicine, University of Ljubljana, SI-1000 Ljubljana, Slovenia; vesna.cvitkovic-spik@mf.uni-lj.si (V.C.-Š.); eva.ruzic-sabljic@mf.uni-lj.si (E.R.-S.); 4 Department of Veterinary Medicine, Faculty of Agriculture, University of Novi Sad, 21000 Novi Sad, Serbia; aleksandar.potkonjak@polj.uns.ac.rs; 5 Department of Environmental and Plant Protection, Faculty of Agriculture, University of Novi Sad, 21000 Novi Sad, Serbia; aleksandar.jurisic@polj.uns.ac.rs

**Keywords:** tick-borne diseases, Borrelia, Anaplasma phagocytophilum, Coxiella burnetii, Serbia

## Abstract

Ticks carry numerous pathogens that, if transmitted, can cause disease in humans and animals. Research on pathogens transmitted from ticks to humans is essential for improving public health strategies against tick-borne diseases (TBDs). In this study, *Ixodes ricinus* ticks found on humans were collected and examined between 2019 and 2024. This study is about the molecular characteristics of tick-borne pathogens (TBPs) in the region of northern Serbia, including *Borrelia burgdorferi* sensu lato (s.l.), *Anaplasma phagocytophilum*, and *Coxiella burnetii*. We identified *B. burgdorferi* s.l. as the most prevalent in ticks (20.45%). Molecular analysis identified two genospecies, *B. afzelii* and *B. burgdorferi* s.s., indicating genetic diversity among *Borrelia* spirochetes. *A. phagocytophilum* was detected in ticks with a prevalence of (1.62%), while *C. burnetii* was not found in any of the ticks. Our findings highlight the necessity of monitoring tick pathogens in ticks removed from humans. Serbia is an endemic region for some tick-borne diseases, such as Lyme disease. Regular surveillance of tick populations, with molecular identification of pathogens, offers insight into transmission dynamics, allowing for monitoring and public health interventions to be created if needed due to increased risk.

## 1. Introduction

Ticks are blood-feeding ectoparasites and vectors of tick-borne pathogens (TBPs) that are important in human and veterinary medicine [1,2,3]. Questing ticks collected from natural habitats carry pathogens, but human-biting ticks offer an insight into the pathogens to which humans are directly exposed. *Ixodes ricinus* is the main vector of Lyme borreliosis in Europe but also can transmit other pathogens such as *Anaplasma phagocytophilum* and *Coxiella burnetii*. So far, we know that Serbia is an endemic area for some tick-borne infections [4]. The main factors influencing geographic spread of ticks can be classified as environmental (e.g., changes in micro- and macroclimate, migration of animals who are natural source of blood meal for ticks) and anthropogenic (e.g., professional and leisure activities, urbanization, traveling, etc.), leading to changes in the prevalence of tick-borne diseases (TBD) [5,6,7,8,9,10,11,12,13]. Influence of the named factors have, so far, contributed to an increased prevalence of TBDs in Serbia, where previous studies pointing out *B. burgdorferi* s.l. as a predominant TBP [14]. Those studies found *I. ricinus* ticks in Serbia infected with *B. burgdorferi* s.l., *A. phagocytophilum*, and *Francisella tularensis* in 26.7% of the infected ticks. Analyses of human-biting ticks present a possibility of determining tick-borne pathogens which could represent a risk for human health. Other studies have also found the infection rate of *B. burgdorferi* s.l. to be at 22.12% in ticks, with a seroprevalence of 25.81% seroprevalence in dogs from Serbia [15]. Further studies have confirmed this by identifying a variety of *Borrelia* genospecies such as *B. afzelii*, *B. garinii*, *B. spielmanii, B. lusitaniae, B. valaisiana; B. miyamotoi* was also identified later on, further emphasizing the genetic diversity of TBPs in the region [16,17]. However, the real prevalence and molecular characteristics of TBPs in ticks removed from humans are underexplored. In addition to *Borrelia* spp., other tick-borne pathogens that are present in Serbia and may represent a health risk are *A.phagocytophilum* and *C. burnetii*. Human anaplasmosis is not very often reported in Serbia, but studies have shown that people bitten by ticks are exposed to *Anaplasma* spp., with rare cases of subclinical bacteremia [9,17,18]. *Coxiella burnetii* has been detected in questing ticks and Q-fever disease remains a reportable disease in Serbia as endemic region. Human cases are reducing in incidence, but are still being reported, and animal cases are continually monitored [19,20]. According to the official report, “Annual report on infection diseases in the Republic of Serbia for 2021”, the incidence rate of Q-fever in humans in Serbia is on the decline, with the last cases reported in 2019, the incidence rate being 0.4/100,000 residents. The most widespread outbreak of Q-fever happened in 2013 when 89 cases were registered within four separate events [21]. All three mentioned diseases are constantly present among animals in Serbia, with or without clinical symptoms [15,20,22,23,24]. This means that the pathogens are circulating between hosts and vectors, representing a risk for possible infection.

This study aims to investigate the molecular characteristics of *B. burgdorferi* s.l., *A. phagocytophilum*, and *C. burnetii* in *I. ricinus* ticks removed from humans during the period of 2019–2024. By focusing on human-biting ticks, this research tries to provide information on the possible transmission of mentioned pathogens relevant for public health and possible risk.

## 2. Materials and Methods

### 2.1. Study Area and Tick Collection

**Figure 1:**
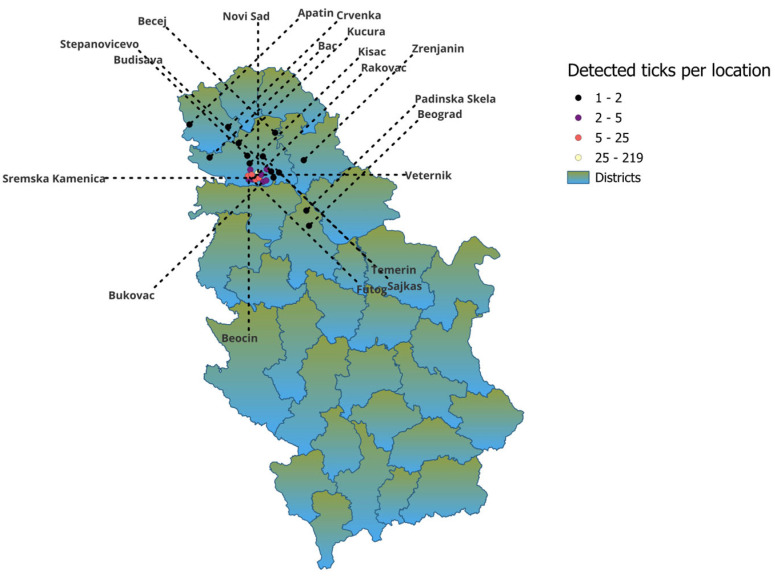
Distribution of collected ticks per location.

The study was conducted in the northern region of Serbia. Medical and veterinary institutions invited and encouraged people to report tick bites throughout the year. Human-biting *Ixodes ricinus* ticks were collected over a 6-year period (2019–2024) from patients seeking medical care in different healthcare facilities. Ticks were collected mostly from the city of Novi Sad area and from other 24 cities in the northern region of Serbia. Detailed distribution information is shown in Table 1 and Figure 1. Patients visited clinics after a tick bite and asked for a tick removal. After the removal of ticks by medical staff, they were placed in labeled plastic tubes or small bottles in medical institutions and then sent to the laboratory of the Scientific Veterinary Institute of “Novi Sad” for analysis.

### 2.2. Morphological Identification of Ticks

In the laboratory of the Scientific Veterinary Institute “Novi Sad”, ticks were identified by species, sex, and life stage based on morphological features using standard taxonomic keys described by Estrada-Pena et al. [25]. The collection locations and number of ticks were recorded. The total number of collected ticks was *n* = 308. Tick samples were stored at −20 °C prior to the molecular analysis.

### 2.3. Sample Preparation and DNA Extraction

Prior to DNA extraction, ticks were washed in 70% ethanol and distilled water. Ticks were then homogenized using the TissueLyser LT (Qiagen, Germantown, MD, USA)-50 Hz, for 3 min. DNA was extracted using the PureLink™ Genomic DNA Mini Kit (Invitrogen, Waltham, MA, USA, REF K182002), following the manufacturer’s instructions. During incubation at 56 °C, for a total of two hours, samples were processed in the TissueLyser LT every 30 min. Extracted DNA was either used immediately for real-time PCR or stored at −20 °C for later analysis.

### 2.4. Molecular Analysis

#### 2.4.1. Real-Time PCR

All ticks were tested for *Borrelia burgdorferi* s.l., *Anaplasma phagocytophilum*, and *Coxiella burnetii* using real-time PCR (Mx3005P^®^ QPCR System, Agilent, Santa Clara, CA, USA). The detection of target pathogens was performed using the following commercial kits: BactoReal^®^ Kit *Borrelia burgdorferi* s.l. (Ingenetix GmbH, Vienna, Austria), *A. phagocytophilum* Genesig Standard Kit (Primerdesign Ltd., Eastleigh, UK), and BactoReal^®^ Kit *C. burnetii* (Ingenetix GmbH, Vienna, Austria).

#### 2.4.2. Nested PCR for Molecular Identification of *Borrelia* spp.

Samples that tested positive for *Borrelia* in real-time PCR were further analyzed at the Laboratory for Diagnostics of Borreliosis and Leptospirosis, Institute of Microbiology and Immunology, Faculty of Medicine, University of Ljubljana, Slovenia. The analysis was conducted for conformation, using the LightMix Kit *Borrelia* spp. EC (TIB MOLBIOL, Berlin, Germany), which detects and identifies *Borrelia* genomic DNA by amplifying a 160 bp fragment of the *ospA* gene. Species differentiation was performed through melting curve analysis on a LightCycler480 II (Roche, Basel, Switzerland).

### 2.5. Molecular Typing of Borrelia *spp.*

Molecular typing was performed on *Borrelia*-positive samples using the *ospC* gene, located on a plasmid. A 617 bp region of the *ospC* gene was amplified using primers OC6(+) and OC623 [26,27] with the Q5^®^ High-Fidelity PCR Kit (New England BioLabs, Ipswich, MA, USA), annealing at 54 °C. Primer sequences and annealing temperatures are shown in Table 2. Amplified products were sequenced using Sanger sequencing (Macrogen Europe, Amsterdam, The Netherlands). Sequences were processed using the Staden package [28] and submitted to GenBank (accession numbers: OR735158–OR735165).

### 2.6. Phylogenetic Analysis

To assess the identity and genetic diversity of the *Borrelia* species identified in this study, obtained *ospC* sequences were analyzed using the Basic Local Alignment Search Tool (BLAST; https://blast.ncbi.nlm.nih.gov/Blast.cgi, accessed on 3 March 2025). In the next step, sequences were aligned using the MUSCLE algorithm. Based on the lowest Bayesian Information Criterion and corrected Akaike Information Criterion, General Time Reversible + Gamma + Invariant Sites (GTR + G + I) model was used to construct a phylogenetic tree. An unrooted phylogenetic tree was constructed to assess the genetic relatedness and genotype clustering of *Borrelia* sp. as the analysis focused on grouping species rather than evolutionary direction. The evolutionary history was inferred using the Maximum Likelihood (ML) method with complete deletion option and bootstrap set at 1000. These analyses and the phylogenetic tree construction were performed using the MEGA 11 software [29].

### 2.7. Statistical Analysis

The Kruskal–Wallis test was used to evaluate differences in pathogen prevalence across the study period. Results were considered statistically significant at *p* < 0.05.

## 3. Results

Out of 308 *I. ricinus* ticks removed from humans, 111/308 were nymphs (36.04%) and 197/308 were adult females (63.96%), while no larvae were detected. *Borrelia* spp. was detected in 56 ticks (17.8%) by real-time PCR. Molecular identification using the LightMix Kit *Borrelia* spp. EC (TIB MOLBIOL) was used to confirm the presence of *B. afzelii* in samples. *Borrelia afzelii* was identified as a sole genospecies in one group. *Borrelia burgdorferi* s.s. was also confirmed by *ospC* genotyping. Genotyping revealed seven successful partial *ospC* gene sequences of *B. afzelii* and *B. burgdorferi* s.s.

*Anaplasma phagocytophilum* was present in only five samples (1.62%; one nymph and four adult females), while *C. burnetii* was not found in any of the ticks. No co-infections were found among the ticks tested.

**Figure 2:**
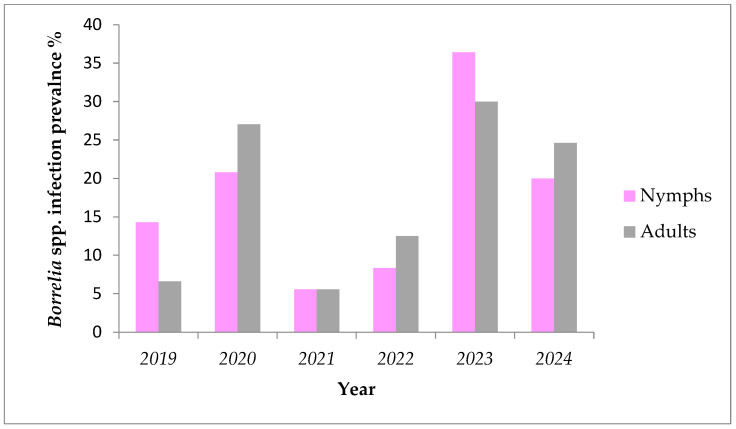
Prevalence of *Borrelia* spp. in ticks removed from humans by development stage during the period of 2019–2024.

During a six-year period (2019–2024) fluctuations were found in prevalence of *Borrelia* spp. in nymphs and adult females removed from humans (Figure 2 and Table 3). The lowest prevalence for both nymphs and adults was registered in the year 2018 (3.44% and 7.69%, respectively), while highest values were observed in 2020 (25% and 33%, respectively). These variations were not statistically significant (Kruskal–Wallis, *p* > 0.05). The numbers of ticks positive for *A. phagocytophilum* over the years are also shown in Table 3.

**Figure 3:**
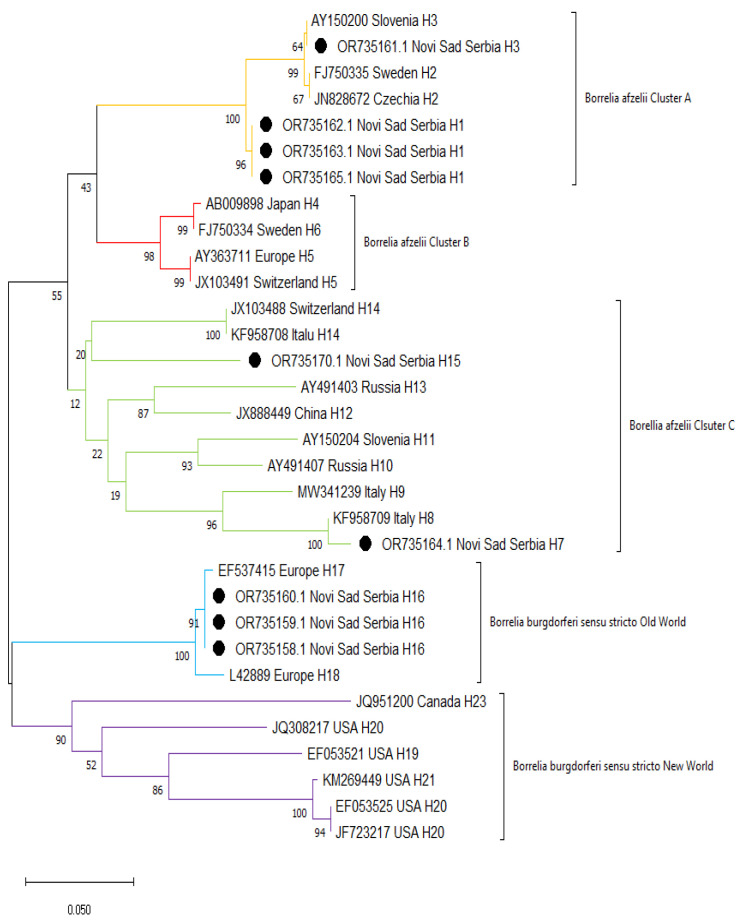
Maximum likelihood phylogenetic tree of *ospC* sequences from *Borrelia* spp. Sequences from this study are marked with black dots.

The results of sequencing of *ospC* gene confirmed species affiliation of the tested samples to *B. burgdorferi* s.s. and *B. afzelii*. The *B. afzelii* sequences obtained in the current study clustered in 2 groups (clusters A and C) together with other sequences reported previously, mainly from Europe (Figure 3). Similarly, *B. burgdorferi* s.s. sequences from Serbia obtained in this study formed a cluster with other European sequences (Old World), whereas North American sequences formed a separate cluster (New World).

## 4. Discussion

Prevalence of *B. burgdorferi* s.l., was reported earlier throughout Serbia as 25–28% in questing *I. ricinus* ticks, collected from vegetation and dogs [30,31]. Also, the similar percentage was found in ticks collected from vegetation and dogs in different parts of a northern Serbian province (Vojvodina) [4,15]. Exposure to *B. burdgorferi* was reported in dogs from the northern part of Serbia. Also, exposure to *B. burgdorferi* s.l., and development of Lyme disease is described in Serbian residents [18,32]. These findings show that both dogs and humans in Serbia have been in contact with the pathogen, with confirmed cases of Lyme borreliosis, indicating that this region is an endemic region for Lyme borreliosis [15,18,33]. There is no official estimation of the national Lyme disease incidence, because the disease stopped being reportable in 2016, regardless of the endemic nature of the disease. In our study, *B. burgdorferi* s.l. was detected in 17.8% of *I. ricinus* ticks removed from humans, which is consistent with reports from 2020 and 2021 [9,18]. Interestingly, higher prevalence rates were recorded in 2019, reaching 33.3% [34]. This might be due to a dynamic pattern of pathogen circulation, influenced by environmental factors, tick population dynamics or host availability. Fluctuations in tick pathogen presence in ticks could not be compared to fluctuations of Lyme borreliosis cases in humans, due to the fact that Lyme borreliosis has been an unreportable disease in Serbia since 2016. That is also one of the reasons for this study—to highlight the importance of disease reporting in following up on the occurrence and potential risk at present and in the future. Genotyping analysis confirmed the presence of both *B. afzelii* and *B. burgdorferi* sensu stricto (*s.s.*) in the collected ticks. *Borrelia afzelii* was found to be the sole genospecies in certain samples. Phylogenetic analysis further demonstrated that Serbian *B. burgdorferi s.s.* sequences clustered with other European strains, forming a group that is distinct from North American variants. This highlights regional genetic similarities that may have implications for Lyme borreliosis epidemiology and pathogenicity. In addition to *Borrelia*, our study detected *Anaplasma phagocytophilum* in 1.62% of ticks taken from humans. This finding aligns with previous studies in Serbia, where *A. phagocytophilum* prevalence in ticks taken off humans, ranged from 6% in 2019 to 9.68% in 2020 and 3.95% in 2021 [9,17,18]. While reports of canine *Anaplasma* infections in Serbia remain scarce [17], seroprevalence studies have detected *Anaplasma*-reactive IgG in 2.45% of cattle (with immunofluorescence test) [23] and 26.1% of dogs (with immunochromatography test) [22]. The presence of *A. phagocytophilum* in Serbian ticks and animals suggests that this pathogen is actively circulating among local vectors and animal hosts. Unlike previous study on questing ticks from Serbia [19], our study did not detect *Coxiella burnetii* in any ticks removed from humans. It could be because the tick bites occurred in urban areas or out-of-the-city green areas, and not on pastures or places near cattle/sheep. The absence of *C. burnetii* in tick samples suggests that while the disease is circulating in the environment, tick infections remain rare or sporadic, and Q-fever is transferred among animals in other ways of infection. Q-fever is definitely present in Serbia among ruminants and sheep [20,24] but as there are other ways of spreading infection apart from tick bites, it could be concluded that the disease among humans is not—or is rarely—spread by ticks. Maybe the result would be different if the ticks were taken off sheep or cattle. The prevalence of *B. burgdorferi* s.l. in ticks that bite humans shows significant variation across the region. In Romania, studies have found *B. burgdorferi* s.l. in 3% of ticks and *A. phagocytophilum* in 1.3% [35]. Similar results were obtained in a study carried out in Cluj-Napoca—a region known for Lyme disease. The authors of this study reported a *B. burgdorferi* s.l. prevalence of 12.64% [36,37]. Higher prevalence rates were observed in Sarajevo Canton, Bosnia and Herzegovina, where *B. spielmanii* and *B*. *lusitaniae* were found in 66.7% of ticks that infest humans, as determined by nested PCR [38]. These differences across the region may be due to variations in tick density, the availability of hosts, climate conditions, and diagnostic methods. These results highlight the need for coordinated surveillance strategies throughout Southeastern Europe. A key limitation of our study is that samples are mainly from the northern part of Serbia, which may not accurately represent pathogen distribution across the entire country, but does represent the endemic region in Serbia. Additionally, we only checked ticks for *Borrelia*, *Anaplasma*, and *Coxiella,* excluding other tick-borne pathogens such as *Rickettsia* and *Babesia*. These three pathogens were the focus of this study, because of their presence in animals (and humans). Future research that includes sampling in different regions of Serbia and whole-genome sequencing could offer a more comprehensive understanding of pathogen diversity and evolution. The variety of *Borrelia* strains and the presence of *A. phagocytophilum* underscore the importance of continued surveillance and public health awareness.

## 5. Conclusions

The presence of tick-borne pathogens in ticks depends on different factors, such as environmental factors, vector distribution, human exposure patterns, etc. Our findings confirm a notable prevalence of *Borrelia* spp. in human-biting ticks, with fluctuations observed over the study period, though without statistical significance. Molecular analysis identified *B. afzelii* and *B. burgdorferi* s.s., indicating genetic diversity among *Borrelia* strains in Serbia. *Anaplasma phagocytophilum* was detected at a low frequency meaning that further research is needed and collection of ticks from different kinds of animals including wild animals. *Coxiella burnetii* was absent, potentially due to its low presence in ticks, and also due to it rarely being transferred by ticks to either humans or animals.

These results reinforce previous reports that Serbia is an endemic region for some tick-borne diseases, highlighting the need for public health measures. The absence of standardized case definitions and diagnostic guidelines in combination with not reporting of the disease, further complicates disease surveillance and management. These results also show how collaboration between environmental, medical and veterinary disciplines can contribute to greater results and knowledge, packed into a One Health approach.

This study provides valuable molecular insights into pathogens in ticks removed from humans, despite limited sample not fully capturing the epidemiological landscape across Serbia. Expanding surveillance efforts, integrating molecular and epidemiological data, and developing targeted control strategies will be essential for looking at the growing public health problem with tick-borne diseases in Serbia.

## Figures and Tables

**Table 1:** Distribution of collected ticks.

City	Number of Ticks
Novi Sad	219
Temerin	1
Kac	4
Rakovac	4
Ledinci	6
Beočin	4
Bukovac	3
Sremska kamenica	25
Stepanovicevo	1
Futog	7
Becej	1
Rumenka	4
Budisava	1
Sremski Karlovci	3
Beograd	1
Kovilj	2
Apatin	1
Veternik	8
Crvenka	1
Kisač	2
Bač	1
Kucura	1
Šajkaš	1
Zrenjanin	1
Padinska skela	1
Petrovaradin	5
**Total**	**308**

**Table 2:** Primers for *ospC* PCR Amplification.

Primer	Position in *ospC*	Sequence (5′–3′)	Tm
OC6 (+)	6	AAA GAA TAC ATT AAG TGC GAT ATT	54 °C
OC623 (−)	623	TTA AGG TTT TTT TTG GAC TTT CTG C	54 °C

**Table 3:** Number of positive ticks removed from humans by development stage by year.

Year	Development Stage	*Borrelia* Positive (*n*/Tested)	*Borrelia* Prevalence Per Year (%)	*Anaplasma* Positive (*n*/Tested)	*Anaplasma* Prevalence Per Year (%)
2019	Nymph	2/14	10.34	1/14	6.89
	Adult Female	1/15		1/15	
2020	Nymph	5/24	24.6	0/24	3.28
	Adult Female	10/37		2/37	
2021	Nymph	1/18	5.55	0/18	1.85
	Adult Female	2/36		1/36	
2022	Nymph	2/24	10.41	0/24	0
	Adult Female	3/24		0/24	
2023	Nymph	4/11	32.26	0/11	0
	Adult Female	6/20		0/20	
2024	Nymph	4/20	23.53	0/20	0
	Adult Female	16/65		0/65	
Total	Nymph	19/111	20.45	1/111	1.62
	Adult Female	44/197		4/197	

## Data Availability

The data presented in this study are available on request from the corresponding author.

## References

[1] Sonenshine D.E. (2018). Range Expansion of Tick Disease Vectors in North America: Implications for Spread of Tick-Borne Disease. Int. J. Environ. Res. Public Health.

[2] De La Fuente J., Antunes S., Bonnet S., Cabezas-Cruz A., Domingos A.G., Estrada-Peña A., Johnson N., Kocan K.M., Mansfield K.L., Nijhof A.M. (2017). Tick-Pathogen Interactions and Vector Competence: Identification of Molecular Drivers for Tick-Borne Diseases. Front. Cell. Infect. Microbiol..

[3] Migné C.V., Hönig V., Bonnet S.I., Palus M., Rakotobe S., Galon C., Heckmann A., Vyletova E., Devillers E., Attoui H. (2022). Evaluation of two artificial infection methods of live ticks as tools for studying interactions between tick-borne viruses and their tick vectors. Sci. Rep..

[4] Potkonjak A., Gutiérrez R., Savić S., Vračar V., Nachum-Biala Y., Jurišić A., Kleinerman G., Rojas A., Petrović A., Baneth G. (2016). Molecular detection of emerging tick-borne pathogens in Vojvodina, Serbia. Ticks Tick-Borne Dis..

[5] Michael L. (2020). Focus on Common Small Animal Vector-Borne Diseases in Central and Southeastern Europe. Acta Vet. Sciendo.

[6] Potkonjak A., Zekic-Stosic M. (2020). Tick-borne infections of dogs in Serbia: A review of research. Vet. Glas..

[7] Ivanović I., Stošić M.Ž., Sabljić E.R., Kišek T.C., Špik V.C., Popović A., Savić S. (2022). Ecology and prevalence of Borrelia burgdorferi s.l. in Ixodes ricinus (Acari: Ixodidae) ticks. Acta Vet. Hung..

[8] Audino T., Pautasso A., Bellavia V., Carta V., Ferrari A., Verna F., Grattarola C., Iulini B., Pintore M.D., Bardelli M. (2021). Ticks infesting humans and associated pathogens: A cross-sectional study in a 3-year period (2017–2019) in northwest Italy. Parasites Vectors.

[9] Banović P., Piloto-Sardiñas E., Mijatović D., Foucault-Simonin A., Simin V., Bogdan I., Obregón D., Mateos-Hernández L., Moutailler S., Cabezas-Cruz A. (2023). Differential detection of tick-borne pathogens in human platelets and whole blood using microfluidic PCR. Acta Trop..

[10] Gray J.S., Dautel H., Estrada-Peña A., Kahl O., Lindgren E. (2009). Effects of climate change on ticks and tick-borne diseases in Europe. Interdiscip. Perspect. Infect. Dis..

[11] Korotkov Y., Kozlova T., Kozlovskaya L. (2015). Observations on changes in abundance of questing Ixodes ricinus, castor bean tick, over a 35-year period in the eastern part of its range (Russia, Tula region). Med. Vet. Èntomol..

[12] Dantas-Torres F. (2015). Climate change, biodiversity, ticks and tick-borne diseases: The butterfly effect. Int. J. Parasitol. Parasites Wildl..

[13] Voyiatzaki C., Papailia S.I., Venetikou M.S., Pouris J., Tsoumani M.E., Papageorgiou E.G. (2022). Climate Changes Exacerbate the Spread of Ixodes ricinus and the Occurrence of Lyme Borreliosis and Tick-Borne Encephalitis in Europe—How Climate Models Are Used as a Risk Assessment Approach for Tick-Borne Diseases. Int. J. Environ. Res. Public. Heal..

[14] Tomanović S., Radulović Ž., Masuzawa T., Milutinović M. (2010). Coexistence of emerging bacterial pathogens in *Ixodes ricinusticks* in Serbia. Parasite.

[15] Savić S., Vidić B., Lazić S., Lako B., Potkonjak A., Lepšanović Z. (2010). *Borrelia burgdorferi* in ticks and dogs in the province of Vojvodina, Serbia. Parasite.

[16] Potkonjak A., Kleinerman G., Gutiérrez R., Savić S., Vračar V., Nachum-Biala Y., Jurišić A., Rojas A., Petrović A., Ivanović I. (2016). Occurrence of Borrelia burgdorferi Sensu Lato in Ixodes ricinus Ticks with First Identification of Borrelia miyamotoi in Vojvodina, Serbia. Vector-Borne Zoonotic Dis..

[17] Banović P., Díaz-Sánchez A.A., Galon C., Simin V., Mijatović D., Obregón D., Moutailler S., Cabezas-Cruz A. (2021). Humans infested with Ixodes ricinus are exposed to a diverse array of tick-borne pathogens in Serbia. Ticks Tick Borne Dis..

[18] Banović P., Díaz-Sánchez A.A., Simin V., Foucault-Simonin A., Galon C., Wu-Chuang A., Mijatović D., Obregón D., Moutailler S., Cabezas-Cruz A. (2022). Clinical Aspects and Detection of Emerging Rickettsial Pathogens: A “One Health” Approach Study in Serbia, 2020. Front. Microbiol..

[19] Tomanović S., Chochlakis D., Radulović Ž., Milutinović M., Ćakić S., Mihaljica D., Tselentis Y., Psaroulaki A. (2013). Analysis of pathogen co-occurrence in host-seeking adult hard ticks from Serbia. Exp. Appl. Acarol..

[20] Debeljak Z., Medić S., Baralić M., Andrić A., Tomić A., Vidanović D., Šekler M., Matović K., Vasković N. (2018). Clinical, epidemiological and epizootic features of a Q fever outbreak in the border region between Serbia and Montenegro. J. Infect. Dev. Ctries..

[21] Jovanović V. (2021). Annual Report on Infectious Diseases in the Republic of Serbia for 2021. Belgrade. https://www.batut.org.rs/index.php?content=2523.

[22] Filipović M.M.K., Beletić A.D., Božović A.V.I., Milanović Z., Tyrrell P., Buch J., Breitschwerdt E.B., Birkenheuer A.J., Chandrashekar R. (2018). Molecular and Serological Prevalence of *Anaplasma phagocytophilum*, *A. platys, Ehrlichia canis, E. chaffeenses, E. ewingii, Borrelia burgdorferi, Babesia canis, B. gibsoni* and *B. vogeli* among Clinically Healthy Outdoor Dogs in Serbia. Vet. Parasitol. Reg. Stud. Rep..

[23] Vasić A., Nieder M., Zdravković N., Bojkovski J., Bugarski D., Pavlović I., Silaghi C. (2018). Tick infestation and occurrence of Anaplasma phagocytophilum and piroplasms in cattle in the Republic of Serbia. Parasitol. Res..

[24] Zutic J., Vojinovic D., Stanojevic S., Kureljusic B., Milicevic V., Kureljusic J., Spalevic L. (2020). Seroprevalence of Coxiella burnetii in cattle in the Belgrade epizootiological area. Biotechnol. Anim. Husb..

[25] Estrada-Peña A., Bouattour A.J.L.C., Camicas J.L., Walker A.R. (2004). Ticks of Domestic Animals in the Mediterranean Region. A Guide to Identification of Species.

[26] Wang I.-N., E Dykhuizen D., Qiu W., Dunn J.J., Bosler E.M., Luft B.J. (1999). Genetic Diversity of ospC in a Local Population of Borrelia burgdorferi sensu stricto. Genetics.

[27] Pearson P., Skaltsis O., Luo C.-Y., Xu G., Oppler Z., Brisson D., Rich S.M. (2022). A Borrelia burgdorferi outer surface protein C (OspC) genotyping method using Luminex technology. PLoS ONE.

[28] Staden R., Beal K.F., Bonfield J.K. (1998). The Staden Package. Bioinformatics Methods and Protocols.

[29] Tamura K., Stecher G., Kumar S. (2021). MEGA11: Molecular Evolutionary Genetics Analysis Version 11. Mol. Biol. Evol..

[30] Milutinović M., Masuzawa T., Tomanović S., Radulović Ž., Fukui T., Okamoto Y. (2008). Borrelia burgdorferi sensu lato, Anaplasma phagocytophilum, Francisella tularensis and their co-infections in host-seeking Ixodes ricinus ticks collected in Serbia. Exp. Appl. Acarol..

[31] Bogunović D., Stević N., Sidi-Boumedine K., Mišić D., Tomanović S., Kulišić Z., Magaš V., Radojičić S. (2018). Molecular Evidence of Q Fever Agent Coxiella Burnetii in Ixodid Ticks Collected from Stray Dogs in Belgrade (Serbia). Acta Vet. Brno..

[32] Banović P., Čapo I., Ogorelica D., Vranješ N., Simin V., Lalošević D. (2019). Mysterious path of *Borrelia spielmanii*: Spreading without morphological alteration of collagen type I and IV. Futur. Microbiol..

[33] Simin V., Lalosevic D., Mijatovic D., Tomanovic S., Miljevic M., Cabrilo B., Bogdan I., Banovic P. (2020). Borellia burgdorferi infection in removed ticks and anti-borrelia antibodies in infested patients admitted to the Pasteur institute, Novi Sad. Vet. Glas..

[34] Banovic P. (2022). Early-Stage Diagnosis and Risk Factors for Tick Infestation and Development of Lyme Borreliosis in Residents of South Bačka District. Ph.D. Thesis.

[35] Anderson M.L. (2007). Infectious causes of bovine abortion during mid- to late-gestation. Theriogenology.

[36] Kalmár Z., Dumitrache M.O., D’amico G., Matei I.A., Ionică A.M., Gherman C.M., Lupșe M., Mihalca A.D. (2020). Multiple Tick-Borne Pathogens in Ixodes ricinus Ticks Collected from Humans in Romania. Pathogens.

[37] Maiwald M., Oehme R., March O., Petney T.N., Kimmig P., Naser K., Zappe H.A., Hassler D., Doeberitz M.V.K. (1998). Transmission risk of *Borrelia burgdorferi* sensu lato from *Ixodes ricinus* ticks to humans in southwest Germany. Epidemiol. Infect..

[38] Lasić L., Ušanović L., Ćakić S., Hanjalić J., Stroil B.K. (2020). First molecular detection of Borrelia burgdorferi in Ixodes ricinu ticks collected from humans in the Sarajevo Canton. Syst. Appl. Acarol..

